# Comparisons of longitudinal speech outcomes in children born with cleft palate ± cleft lip with and without additional conditions at 5 and 10 years –a registry study

**DOI:** 10.1186/s12887-026-06841-3

**Published:** 2026-04-11

**Authors:** Ann Malmenholt, Christina Persson, Kristina Klintö

**Affiliations:** 1 Division of Speech and Language Pathology, Department of Clinical Science, Intervention and Technology (CLINTEC), Karolinska Institutet, Stockholm, Sweden; 2 Speech and Language Pathology, Medical Unit Allied Health Professionals, Karolinska University Hospital, Stockholm, Sweden; 3 Institute of Neuroscience and Physiology, Department of Health and Rehabilitation, Speech and Language Pathology Unit, Sahlgrenska Academy, University of Gothenburg, Gothenburg, Sweden; 4 Region Västra Götaland, Department of Otorhinolaryngology, Sahlgrenska University Hospital, Gothenburg, Sweden; 5 Division of Speech Language Pathology, Phoniatrics and Audiology, Department of Clinical Sciences in Lund, Lund University, Lund, Sweden; 6 Division of Speech Language Pathology, Department of Otorhinolaryngology, Skåne University Hospital, Malmö, Sweden

**Keywords:** Cleft lip and palate, Speech, Syndromes, Additional malformations, Intellectual disability

## Abstract

**Background:**

Speech outcome differences in children with cleft palate ± cleft lip (CP ± L) with ( +) and without (-) additional conditions (i.e., diagnosed syndromes, additional malformations, and intellectual disabilities) are not fully explored. The Swedish cleft lip and palate (CLP) registry enables a longitudinal approach. Thus, the purpose was to compare longitudinal speech outcomes in children with different cleft types, with and without additional conditions, at ages 5 and 10 years, based on registry data.

**Methods:**

Included were all children with oral clefts involving the palate born in Sweden 2009–2012, with speech registrations at both 5 and 10 years in the Swedish CLP registry (*n* = 404): 16 cleft soft palate (SP) + , 37 SP-, 67 cleft soft and hard palate (SHP) + , 88 SHP-, 19 unilateral cleft lip palate (UCLP) + , 116 UCLP-, 15 bilateral cleft lip palate (BCLP) + and 46 BCLP-. The speech outcomes percentage of consonants correct, percentage of nonoral speech errors, velopharyngeal competence, and results of the Intelligibility in Context Scale were dichotomized. For group comparisons, Pearson’s chi-square test and Fisher’s exact test were performed.

**Results:**

No significant differences were found between the UCLP + /UCLP- and BCLP + /BCLP- groups. In the SP group, a significantly lower proportion of participants with additional conditions than without had age-appropriate consonant production at age 5 (SP + 33.3%; SP- 79.4%), sufficient velopharyngeal function at age 10 (SP + 73.3%; SP- 97.3%), and always intelligible speech at age 10 (SP + 33.3%; SP- 78.6%). In the SHP group, a significantly lower proportion of participants with additional conditions than without had age-appropriate consonant production at age 5 (SHP + 60.6%; SHP- 82.1%) and 10 (SHP + 73.8%; SHP-95.5%), sufficient velopharyngeal function at age 5 (SHP + 82.1%; SHP- 95.5%), and always intelligible speech at age 10 years (SHP + 51.7%; SHP- 83.8%).

**Conclusions:**

For all cleft types with and without additional conditions and for all measured speech outcomes, speech improved between 5 and 10 years of age. Speech results were poorer for children with SP + and SHP + at both 5 and 10 years of age. The results highlight the need to consider heterogeneity within cleft subgroups when planning speech improving interventions.

## Background

Despite early surgical treatment, cleft palate may have a negative impact on speech development [1–6]. Speech is a complex function. Besides well-functioning structure and speech motor skills, typical speech development requires good hearing [7] and also phonological skills, i.e., the language-specific contrastive use of speech sounds and the phonotactic rules for combining them [8]. In some cases, additional conditions, such as, syndromes, additional malformations, and intellectual disabilities may occur in children with cleft palate and can affect speech and language development [9]. These genetic conditions might have an impact on several patientrelated factors such as deviant pharyngeal dimensions (depth and width), muscle hypotonia [10] and higher prevalence of abnormal middle ear status and elevated hearing thresholds [11]. Children with oral clefts and additional conditions are therefore usually excluded from studies of speech [1–6]. Thus, studies of speech in children with oral clefts and additional conditions are few, and knowledge of the extent to which additional conditions affect speech in children with cleft palate is limited. There is a need for separate studies of subgroups of cleft palate with and without additional conditions to ensure a more comprehensive understanding of how different conditions may interact with speech development.

Oral clefts are the most common craniofacial anomalies, with reported prevalence rates between 0.33 and 2.18 per 1000 births varying across geographical regions [12]. Reports on the proportions of children with oral clefts with additional conditions are affected by different methodologies and the availability of resources and techniques used to assess genetic and medical conditions, both globally and over time [13]. A finding true for all studies investigating the prevalence of additional conditions in children born with oral clefts is that a cleft lip is least associated with additional malformations, chromosomal or nonchromosomal anomalies and syndromes (e.g., [12, 14–16]). The highest prevalence of additional conditions is reported in children with an isolated cleft palate (CP), which includes cleft soft palate (SP) and cleft soft and hard palate (SHP) (e.g., [12, 15, 16]). The prevalence reported for children born with unilateral or bilateral cleft lip and palate (CLP) is somewhat lower than that reported for those born with CP. Studies investigating the prevalence of additional conditions in European participants with cleft lip and/or CP, with a total number of 4791 individuals, some analysing populations over several decades [12, 14, 15, 17], reported additional conditions in 24–37% of children with CLP [12, 14, 15] and 34–52% of children with CP [9, 12, 15, 17]. In a study of Japanese participants born with oral clefts between 2011 and 2014 (*n* = 248), differences in proportions between children with additional conditions within cleft subgroups CP and CLP were smaller: 26.9% for CLP and 31% for CP [16]. Intellectual disability is more rarely reported in studies on additional conditions in children with oral clefts. In one Swedish study investigating 343 children with CP, it was found in 18% of participants [17]. An Australian study reported intellectual disability in 8.6% of 1034 children with orofacial clefts [18].

The Swedish national quality registry for cleft lip and/or CP (CLP registry) provides information on diagnosed syndromes and/or additional malformations, typically reported by surgeons when children are enrolled in the registry just days after birth and updated coherently [19]. In addition, information on diagnosed intellectual disability is registered continuously. Speech outcome is reported by speech-language pathologists at fixed time points and collected during speech follow-ups at ages 18 months, 5, 10, 16 and 19 years. For more information about the variables within the Swedish CLP registry, see Klintö et al. [19].

Studies on speech in children with cleft palate with or without cleft lip (CP ± L) usually aim for groups that are as homogeneous as possible. Since additional conditions (+), i.e., diagnosed syndromes, additional malformations, and intellectual disabilities may affect speech and language development in children with CP ± L [9], children with CP ± L + mostly are excluded from large studies reporting speech outcomes. This is, for example, the case in the Scandcleft randomized trials [1–3], the TOPS trial [4], the Cleft Care UK study [5], and a national cohort study from New Zealand [6]. A subgroup of children with cleft palate sometimes included (e.g., [20, 21]) and sometimes excluded in studies on CP (e.g., [4]) are children with Robin sequence (RS). The sequence is pathogenically heterogeneous, and the characteristic micrognathia found in all children results in glossoptosis and upper airway obstruction. The majority also present with an often wide, U-shaped cleft palate [22–24]. The heterogeneous group of children with RS can be further classified into subgroups: isolated RS, i.e., nonsyndromic RS, syndromic RS, and, in some studies, RS plus indicating associated conditions but no identifiably specific syndrome [22]. The prevalence of different RS subgroups varies but has historically been reported as 50/50 for isolated RS and syndromic RS [23]. Two studies from the United States illustrate how differing subclassifications of RS can impact comparisons of groups with and without additional conditions. One study analysing 75 consecutive cases of RS that underwent CP repair from 1990 to 2016 [22] reported 45% for isolated RS, 22% for syndromic RS, and 33% for RS plus. The other study, which reviewed the medical records of children born with RS between 2007 and 2017 (*n* = 234) [25], reported rates of 72% for isolated RS and 28% for syndromic RS.

Several studies have compared speech outcomes in children with CP ± L + and CP ± L without additional conditions (CP ± L-) [9, 24, 26–28]. One Swedish study of one hundred 5-year-olds from one CLP center using the same speech outcomes as the CLP registry revealed no statistically significant differences between groups of children with CP ± L + and CP ± L-, although the average results in the CP ± L + group indicated poorer speech [26]. In a prospective cohort study, the articulation proficiency of 88 Swedish 5-year-olds with CP ± L was evaluated using the percentage of consonants correct (PCC) as an outcome measure [27]. The articulation proficiency of children with additional malformations, such as RS, did not differ significantly from that of children without additional conditions [27]. In addition, a study of 22 German five- to six-year-old children with isolated RS concluded that these children presented good speech development in line with the speech of 22 peers with cleft palate only [28]. There are, on the other hand, studies with contradictory findings. In a study by Hardwicke and colleagues [24], a cohort of 24 British 5-year-olds with RS (3 syndromic RS; 21 isolated RS) had a significantly higher occurrence of hypernasality and cleft speech characteristics than did matched children with cleft palate without RS. Persson et al. [9] investigated a total of 59 Swedish children with isolated cleft palate (22 CP +; 37 CP-) and reported that 5-year-olds with CP- had satisfactory speech at group level, whereas the group of children with CP + did not.

Although several longitudinal speech outcome studies have focused exclusively on a specific type of cleft, e.g., CP [20, 21] or unilateral CLP (UCLP) [29, 30], there are a few longitudinal outcome studies that have included all types of CP ± L [31, 32]. These studies evaluated the speech of children with oral clefts in Swedish [31] and Japanese [32] and included speech outcome measures of articulation and velopharyngeal function at ages 5 to 10. Gradual improvement in speech outcomes over time was reported [31, 32]. To our knowledge, no study including all types of CP ± L has compared longitudinal speech outcomes between children with CP ± L + and children with CP ± L-.

Speech outcome is one of the primary measures for evaluating cleft palate care and reflects both the methods and the timing of surgical interventions as well as the effects of speech therapy over time. The International Consortium of Health Outcome Measures (ICHOM; www.ichom.org) recommends PCC [33] and rating of velopharyngeal competence, using the three-point scale VPC-R [34] as patient-centred perceptually based speech outcomes for CP ± L [35]. Furthermore, the Intelligibility in Context Scale [36] is recommended by ICHOM as a measure of functional intelligibility at approximately 5 and 10 years of age [35]. In the Swedish CLP registry, data on the PCC, velopharyngeal competence, and percentage of nonoral speech errors are registered [19, 37–39], and the reliability of binary quality indicators on the basis of the speech data at ages 5 [37, 38] and 10 years [39] has proven to be satisfactory. In 2016, the Intelligibility in Context Scale [36] was included in the CLP registry.

In summary, to determine whether speech outcomes differ between children with CP ± L + and those with CP ± L-, a systematic survey including a complete cohort of children born with CP ± L is needed. Using the Swedish CLP registry enables a retrospective, longitudinal approach, given that speech outcomes at both 5 and 10 years are available from all six Swedish CLP centres with data from standardized speech assessments. The aim of this study was to compare longitudinal speech outcomes at 5 and 10 years in children with CP ± L + and CP ± L-. We also assessed the occurrence of secondary speech-improving surgery and speech-language therapy. We started from the null hypothesis: there are no differences in speech outcome, occurrence of secondary speech-improving surgery, and speech-language therapy when comparing subgroups of children with oral clefts with and without additional conditions.

## Methods

### Design and setting

CLP registry data were collected and recorded by speech-language pathologists at the six Swedish regional CLP centers for routine speech follow-ups at 5 and 10 years. The dataset from the CLP registry was retrieved via Record Centre South, Lund, Sweden.

### Participants and coverage degree

**Fig. 1:**
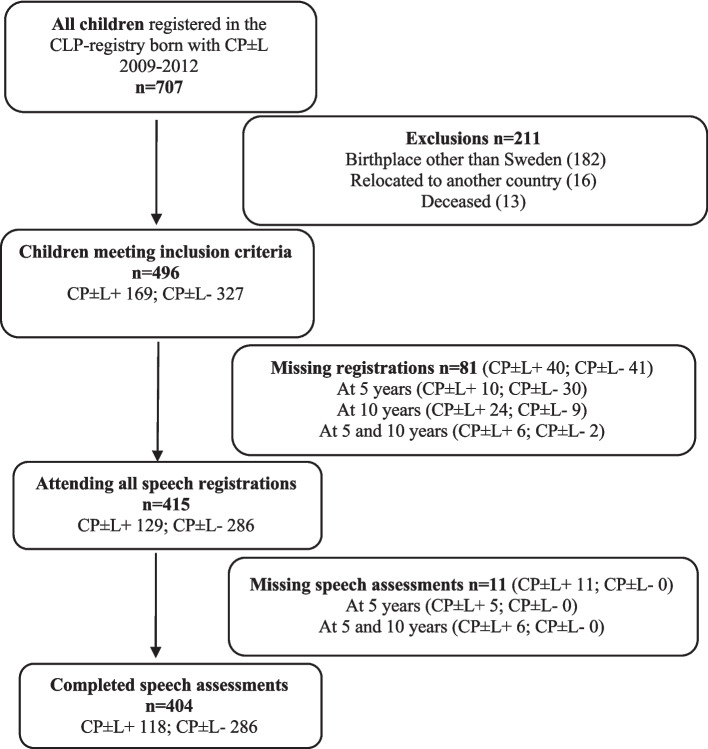
Number of children in the cohort with cleft palate with or without cleft lip (CP ± L), exclusions, missing registrations and missing assessments, presented separately for children with (+) and without (-) syndromes and/or additional malformations and/or diagnosed intellectual disability

CLP registry data from children born from 2009 to 2012 were included. Children who were deceased (*n* = 13), born abroad (*n* = 182), or who had moved abroad before registration at 10 years of age (*n* = 16) were excluded. This resulted in 496 children, as shown in Fig. 1.

The coverage degree for children in the CLP registry born with any type of cleft in Sweden from 2009 to 2014 was 90.7% [40]. The figure was calculated by comparing data of cleft diagnosis (ICD-10 [41]) from the Central patient registry managed by the Swedish National Board of Health and Welfare with data from the CLP registry.

### Registration of background data

At baseline, data were registered, including if children were born in Sweden, cleft morphology, cleft type (ICD-10 diagnosis [41]), presence of syndromes, Robin sequence, and other additional malformations. Syndromes, additional malformations, and intellectual disabilities were also possible to register after diagnosis later in childhood. Information on type of syndrome or additional malformation is not available in the CLP registry. For more detailed information, see Klintö et al. [19]. A manual review of the data was performed every year by each CLP team and by the last author before analysis. Suspected missing or faulty registrations, e.g., cleft diagnosis not corresponding to cleft morphology, were validated against medical records and corrected when needed.

### Surgical protocols and data

In cases of a cleft in the lip and alveolus, the child underwent lip-plasty with simultaneous correction of the nasal cartilage at 3 to 6 months of age. The procedures for palatal closure differ between Swedish CLP centers in terms of timing, staging and technique [40]. According to the official surgical protocols, primary palate surgery in one-stage protocols was performed between approximately at 9 to 18 months of age. In two-stage protocols, the soft palate was closed at approximately 6 months of age, and the hard palate was closed at approximately 2 years of age. However, at one CLP center in some cases, the hard palate was closed in more than two stages [40]. In cases of cleft alveolus, the cleft in the alveolar ridge was or should be closed in the mixed dentition at 7 to 11 years of age by a cancellous bone graft harvested either from the iliac crest or the tibia. For more information on cleft palate surgery in Swedish-born children with CP ± L-, see Klintö et al. [40].

Surgical data were recorded continuously and coded according to the Swedish National Board of Health and Welfare’s classification of health interventions [42]. The reporting degree of cleft-related surgeries, performed until 2022 for all children born from 2009 to 2012 in the CLP registry, was assessed by comparing the number of cleft-related surgical intervention codes in the CLP registry for each individual with the number of surgical intervention codes for each individual in the Central patient registry. The reporting degree of cleft-related surgeries for children born from 2009 to 2012 was 94.6%.

The outcome measure secondary speech-improving surgery included rerepair of the palate after the age of 3 years, pharyngeal flap, buccal flap, and plastic surgery of the pharynx (e.g., sphincter pharyngoplasty). A manual review of the surgical data was performed every year by each CLP team and by the last author before analysis. Suspected missing or faulty registrations, e.g., surgical codes or timing of surgery not conforming with cleft diagnosis, were validated against medical records and corrected when needed.

### Speech assessment and registration of speech data

At 5 years of age ± 6 months and at 10 years ± 12 months, a speech-language pathologist at one of the six CLP centers documented the child’s speech with standardized audio recordings, according to the assessment procedure of the Swedish Articulation and Nasality Test (SVANTE), including single-word naming, sentence repetition, and continuous speech [43]. For Swedish CLP speech-language pathologists introductory training must be completed, and participation in regional and national calibration is mandatory [39]. Listening calibration is a recurring item at the annual meetings of Swedish CLP speech-language pathologists. Calibration among speech-language pathologists within regional CLP teams are scheduled twice a year [39].

In most cases, at 5 years (91.8%), the SVANTE minimum standard set (*n* = 30 words), included in the SVANTE single-word naming test (*n* = 59 words) [43], was used, and in 5.6% of the cases, the word test in the randomized controlled trial Timing of Primary Surgery for Cleft Palate (TOPS) (*n* = 36 words) was used [4]. The three single-word tests were designed to analyse cleft speech characteristics and, according to the same principles, to examine one target consonant per word [44]. At 10 years of age, the SVANTE minimum standard set was used in 1.5% of the cases, and the ordinary SVANTE word test was used in the remaining 98.5%, which was the result of changed routines starting in 2019. In addition, at 10 years of age, the caregivers completed the Intelligibility in Context Scale [36] by answering seven questions in which they estimated how well the child was understood by different people. The scale used ranges from 1 to 5, where 1 corresponds to never being understood and 5 always being understood by different communication partners.

On the basis of the recordings, the speech-language pathologists performed phonetic semi-narrow transcriptions according to the International Phonetic Alphabet [45] for the 59 target consonants in SVANTE, the 30 target consonants in the SVANTE minimum standard set, or the 36 target consonants in the TOPS word set and calculated the PCC and percentage of nonoral speech errors [43]. Based on the transcriptions of target consonants PCC was calculated by dividing the number of correct consonants by the number of elicited consonants.Weak articulation and audible nasal air leakage marked with diachritics were scored as correct in the PCC calculation. Errors that changed the target consonants to another phoneme were scored as incorrect. By dividing the number of non-oral speech errors (glottal and pharyngeal articulation as well as active nasal fricatives) by the number of elicited target consonants the percentage of non-oral speech errors was calculated. As a prerequisite for registration into the CLP registry, at least half of the target consonants were required to have been produced. On the basis of the impressions of hypernasality, audible nasal air leakage and weak articulation in the whole speech material, speech-language pathologists performed an overall rating of perceived velopharyngeal competence on the three-point scale VPC-R, with scale values of ‘competent/sufficient’, ‘marginally incompetent/insufficient’ and ‘incompetent/insufficient’ [43].

In connection with the follow-up assessments at 5 and 10 years of age, the CLP center speech-language pathologists collected information from the caregivers and the local speech-language pathologists about received speech-language therapy. A manual review of the speech data was performed every year by a speech-language pathologist at each CLP center and by the last author before analysis to ensure data correctness. Suspected missing or faulty registrations, e.g., contradictory speech results, were validated against medical records and corrected when needed.

### Dichotomization of speech variables and statistical analysis

For statistical analysis, the speech variables were dichotomized in the same way as the quality indicators in the CLP registry [37–39]. PCC was dichotomized into the outcome measure age-appropriate consonant production (yes/no), defined as ≥ 86% correct consonants at 5 years of age [38] and ≥ 92.3% at 10 years of age [39]. The cut-offs correspond to −2 standard deviations from the mean in norm data of Swedish-speaking 5- and 10-year-olds without CLP [43]. The percentage of nonoral speech errors was dichotomized into without nonoral errors (yes/no), where a maximum of 5% nonoral speech errors was allowed, as a margin of errors [37, 39]. The VPC-R was dichotomized into sufficient velopharyngeal function (yes/no), defined as competent or marginally incompetent velopharyngeal function [37, 39]. Finally, the Intelligibility in Context Scale at 10 years of age was dichotomized into always intelligible speech (yes/no), defined as always being understood by all communication partners [46].

Descriptive data included measures of central tendency and distribution. Since the results were not normally distributed and groups in many cases were small, nonparametric tests were used for group comparisons. For comparisons of age at completed primary palatal surgery, the Mann–Whitney U-test was used. For comparisons of binary outcomes, the Pearson χ^2^ test was used, or Fisher’s exact test when more than 20% of the cells had expected frequencies < 5. Differences in which *p* < 0.05 (two-tailed) were considered significant.

## Results

### Data set

Since the study aimed for longitudinal speech outcomes at 5 and 10 years, children with missing speech registrations within the time span (5 years ± 6 months, 10 years ± 12 months) or missing perceptual speech assessments were excluded (see Fig. 1). This resulted in 404 children with speech data at both 5 and 10 years of age. The number of participants from the six CLP centers varied depending on the CLP centers’geographical area (center 1, *n* = 41; center 2, *n* = 88; center 3, *n* = 97; center 4, *n* = 80; center 5, *n* = 36; center 6, *n* = 62). At age 5, datasets for speech were complete in 92.8% of the 404 cases. Data for age-appropriate consonant production were missing for 13 participants (3.2%), for without nonoral errors for 12 participants (3%), and for sufficient velopharyngeal function for four partcipants (1%). At age 10, datasets for speech were complete in 96.8% of the cases. Data for age-appropriate consonant production were missing for five partcipants (1.2%), for without nonoral errors for five partcipants (1.2%), and for sufficient velopharyngeal function for three participants (0.7%). There were missing data regarding speech-language therapy between 5 and 10 years of age for 18 participants (4.5%) and speech intelligibility at 10 years of age for 61 participants (15.1%).

### Cleft diagnoses and additional conditions

The numbers of children with different cleft types in the CP ± L + and CP ± L- groups are presented in Table 1. They were divided into subgroups according to cleft type: SP, SHP, UCLP, and bilateral CLP (BCLP). Eight children had atypical clefts and were fitted into one of the four cleft subgroup categories on the basis of the expected impact of the cleft on speech function. Consequently, one boy without an additional diagnosis with SP and unilateral cleft lip was included in the SP group, and one girl with intellectual disability with SHP and bilateral cleft lip was included in the SHP group. One boy with another malformation and SP, a unilateral cleft alveolus, and a bilateral cleft lip was included in the UCLP group. In addition, two boys and three girls with SP, unilateral cleft alveolus, and unilateral cleft lip were included in the UCLP group. Table 2 presents the CP ± L + group with information about the different types of additional conditions reported by cleft subgroup.

**Table 1:** Distribution of cleft type and sex

Included children (*n* = 404)	CP ± L + (*n* = 117)			CP ± L- (*n* = 287)			Proportion (%) of children with a specific cleft type with +
	Girls	Boys	Total	Girls	Boys	Total	
SP (*n* = 53)	7	9	16	20	17	37	30.2
SHP (*n* = 155)	37	30	67	59	29	88	43.2
UCLP (*n* = 135)	8	11	19	41	75	116	14.1
BCLP (*n* = 61)	4	11	15	15	31	46	24.6

CP ± L = cleft palate with or without cleft lip, + = with syndromes and/or additional malformations and/or intellectual disabilities, - = without syndromes, additional malformations, and intellectual disabilities, SP = cleft soft palate, SHP = cleft soft and hard palate, UCLP = unilateral cleft lip and palate, BCLP = bilateral cleft lip and palate

**Table 2:** Information on additional conditions in the cleft subgroups

CP ± L + (*n* = 117)	SP (*n* = 16)	SHP (*n* = 67)	UCLP (*n* = 19)	BCLP (*n* = 15)
Isolated Robin sequence (*n* = 38)	4	34		
Robin sequence plus* (*n* = 5) Syndromic Robin sequence** (*n* = 5)	1	4 5		
Associated CP ± L* (*n* = 49) Syndromic CP ± L** (*n* = 20)	7 4	18 6	14 5	10 5

*SP* Cleft soft palate, *SHP* Cleft soft and hard palate, *UCLP* Unilateral cleft lip and palate, *BCLP* Bilateral cleft lip and palate

^*^With other malformations and/or intellectual disability but no diagnosed syndrome

^**^With a diagnosed syndrome

### Age at completed primary palatal surgery, occurrence of secondary speech-improving surgery and speech-language therapy

No significant differences were seen regarding age at completed primary surgery between the cleft subgroups with and without additional conditions, when tested with Mann Whitney test. The Z-values ranged between −2.440 (*p* = 0.150) and −1.024 (*p* = 0.306) (Table 3).

**Table 3:** Age at completed primary palatal surgery in groups with and without additional conditions

Groups	Age in years at completed primary palatal surgery		Z	*p*
	**Median (min–max)**	**Mean (SD)**		
SP + (*n* = 16)	0.96 (0.47–1.57)	0.88 (0.315)	−1.066	0.286
SP- (*n* = 37)	0.6 (0.41–3.34)	0.82 (0.523)		
SHP + (*n* = 67)	1.08 (0.47–2.72)	1.35 (0.604)	−2.440	0.150
SHP- (*n* = 88)	0.99 (0.41–3.09	1.16 (0.622)		
UCLP + (*n* = 19)	1.13 (0.74–3.02)	1.58 (0.742)	−1.024	0.306
UCLP- (*n* = 115)	2.01 (0.48–5.23)	1.78 (0.660)		
BCLP + (*n* = 15)	1.49 (0.96–2.73)	1.61 (0.609)	−1.298	0.194
BCLP- (*n* = 46)	2.02 (0.87–3.51)	1.88 (0.660)		

+ = with syndromes and/or additional malformations and/or intellectual disabilities, - = without syndromes, additional malformations, and intellectual disabilities, SP = cleft soft palate, SHP = cleft soft and hard palate UCLP = unilateral cleft lip and palate, BCLP = bilateral cleft lip and palate

There were no significant differences in the cleft subgroups with and without additional conditions regarding the proportions of children who had their first secondary speech improving surgery before 5 years of age or between 5 and 10 years of age. The χ2 values ranged from 0.073 (*p* = 0.677) to 2.008 (*p* = 0.213) (Table 4).

**Table 4:** Secondary speech improving surgery and speech-language pathologist (SLP) therapy in groups with and without additional conditions

Groups	First secondary speech improving surgery before 5 years				First secondary speech improving surgery between 5 and 10 years			
	n	Yes (%)	χ2	*p*	n	Yes (%)	χ2	*p*
SP +	16	0 (0)	N/A	N/A	16	2 (12.5)	2.008	0.213
SP-	37	0 (0)			37	1 (2.7)		
SHP +	67	5 (7.5)	0.200	0.747	67	6 (9)	0.618	0.533
SHP-	88	5 (5.7)			88	5 (5.7)		
UCLP +	19	2 (10.5)	0.314	0.632	19	10 (8.6)	0.073	0.677
UCLP-	116	8 (6.9)			116	2 (10.5)		
BCLP +	15	1 (6.7)	1.034	0.430	15	6 (13)	0.435	0.676
BCLP-	46	8 (17.4)			46	3 (20)		

**Groups**	**SLP therapy before 5 years**				**SLP therapy between 5 and 10 years**			
	n	Yes (%)	χ2	*p*	n	Yes (%)	χ2	*p*
SP +	16	10 (62.5)	11.354	0.002	16	7 (43.8)	11.291	0.002
SP-	37	6 (16.2)			36	2 (5.6)		
SHP +	67	29 (43.3)	3.136	0.077	64	19 (29.7)	2.613	0.106
SHP-	88	26 (29.5)			82	15 (18.3)		
UCLP +	19	10 (52.6)	0.600	0.438	17	6 (35.3)	0.000	0.993
UCLP-	116	50 (43.1)			113	40 (35.4)		
BCLP +	15	10 (66.7)	0.295	0.741	13	7 (53.8)	0.718	0.515
BCLP-	46	34 (73.9)			45	30 (66.7)		

+ = with syndromes and/or additional malformations and/or intellectual disabilities, - = without syndromes, additional malformations, and intellectual disabilities, SP = cleft soft palate, SHP = cleft soft and hard palate, UCLP = unilateral cleft lip and palate, BCLP = bilateral cleft lip and palate, CP ± L = cleft palate with or without cleft lip, N/A = not applicable

Statistically significant values (<.05) are marked in bold

A significantly higher proportion of children with SP + (62.5%) than SP- (16.2%) had been enrolled in speech-language therapy before 5 years (χ2 = 11.354; *p* = 0.002) and between 5 and 10 years of age (χ2 = 11.291; *p* = 0.002). Between 5 and 10 years of age, 43.8% of those in the SP + group had been enrolled in speech-language therapy, whereas 5.6% of those in the SP- group had been enrolled. No other significant differences were detected between children with and without additional conditions in the different cleft subgroups (Table 4).

### Speech outcomes

A significantly lower proportion of children with SP + (33.3%) than SP- (79.4%) and SHP + (60.6%) than SHP- (82.1%) had age-appropriate consonant production at 5 years of age (Table 5). At 10 years of age, a significantly lower proportion of children with SHP + (73.8%) than SHP- (95.5%) had age-appropriate consonant production. No significant differences between groups were observed regarding the outcome without nonoral errors. A significantly lower proportion of children with SHP + (82.1%) than SHP- (95.5%) had sufficient velopharyngeal function at 5 years of age. At 10 years of age, a significantly lower proportion of children with SP + (73.3%) than SP- (97.3%) had sufficient velopharyngeal function (Table 5). A significantly lower proportion of children with SP + (33.3%) than SP- (78.6%) and with SHP + (51.7%) than SHP- (83.8%) had always intelligible speech at 10 years of age (Table 6). No other significant differences were detected between subgroups with and without additional conditions (Tables 5 and 6).

**Table 5:** Speech outcomes for groups with and without additional conditions at 5 and 10 years

Groups	*Age-appropriate consonants* 5 years				Age-appropriate consonants 10 years			
	n	Yes (%)	χ2	*p*	n	Yes (%)	χ2	*p*
SP +	15	5 (33.3)	9.754	0.002	15	14 (93.3)	0.453	0.498
SP-	34	27 (79.4)			37	36 (97.3)		
SHP +	66	40 (60.6)	8.631	**0.003**	65	48 (73.8)	14.741	** < 0.001**
SHP-	84	69 (82.1)			88	84 (95.5)		
UCLP +	18	5 (27.8)	3.842	0.050	18	13 (72.2)	0.023	1.000
UCLP-	114	60 (52.6)			115	85 (73.9)		
BCLP +	14	4 (28.6)	0.124	0.734	15	5 (33.3)	1.610	0.204
BCLP-	46	11 (23.9)			46	24 (52.2)		

**Groups**	**Without nonoral errors at 5 years**				**Without nonoral errors at 10 years**			
	n	Yes (%)	χ2	*p*	n	Yes (%)	χ2	*p*
SP +	15	12 (80)	4.040	0.079	15	14 (93.3)	0.453	0.498
SP-	34	33 (97.1)			37	36 (97.3)		
SHP +	66	56 (84.8)	3.568	0.059	65	59 (90.8)	2.289	0.170
SHP-	85	80 (94.1)			88	85 (96.6)		
UCLP +	18	15 (83.3)	0.087	0.724	18	17 (94.4)	0.186	0.522
UCLP-	114	98 (86)			115	111 (96.5)		
BCLP +	14	11 (78.6)	1.165	0.347	15	14 (93.3)	1.034	0.430
BCLP-	46	29 (63)			46	38 (82.6)		

**Groups**	**Sufficient VPF 5 years**				**Sufficient VPF 10 at years**			
	n	Yes (%)	χ2	*p*	N	Yes (%)	χ2	*P*
SP +	14	11 (78.6)	2.949	0.120	15	11 (73.3)	7.053	**0.021**
SP-	37	35 (94.6)			37	36 (97.3)		
SHP +	67	55 (82.1)	7.340	0.015	67	61 (91)	1.226	0.331
SHP-	88	84 (95.5)			88	84 (95.5)		
UCLP +	19	15 (78.9)	0.860	0.474	19	18 (94.7)	0.035	1.000
UCLP-	115	100 (87)			116	111 (95.7)		
BCLP +	15	11 (73.3)	0.000	1.000	15	13 (86.7)	0.057	1.000
BCLP-	45	33 (73.3)			44	37 (84.1)		

+ = with syndromes and/or additional malformations and/or intellectual disabilities, - = without syndromes, additional malformations, and intellectual disabilities, SP = cleft soft palate, SHP = cleft soft and hard palate, UCLP = unilateral cleft lip and palate, BCLP = bilateral cleft lip and palate, VPF = velopharyngeal function

Statistically significant values (<.05) are in bold

**Table 6:** Caregiver-reported intelligibility at 10 years in groups with and without additional conditions

Groups	Always intelligible speech at 10 years			
	n	Yes (%)	χ2	*p*
SP +	9	3 (33.3)	6.360	0.036
SP-	28	22 (78.6)		
SHP +	58	30 (51.7)	16.514	** < 0.001**
SHP-	80	67 (83.8)		
UCLP +	12	6 (50)	0.974	0.357
UCLP-	101	65 (64.4)		
BCLP +	13	3 (23.1)	0.489	0.733
BCLP-	42	14 (33.3)		

+ = with syndromes and/or additional malformations and/or intellectual disabilities, - = without syndromes, additional malformations, and intellectual disabilities, SP = cleft soft palate, SHP = cleft soft and hard palate, UCLP = unilateral cleft lip and palate, BCLP = bilateral cleft lip and palate

Statistically significant values (<.05) are in bold

## Discussion

The aim of the present study was to compare longitudinal speech outcomes in children with different cleft types, with and without additional conditions, at ages 5 and 10. We also assessed the occurrence of secondary speech-improving surgery and speech-language therapy. By using national registry data, including all children with speech registrations at 5 and 10 years, born during four consecutive years, we aimed for representativity of results. Speech improved between 5 and 10 years of age for all cleft types with and without additional conditions. In the largest cleft groups with additional conditions, SP + and SHP +, speech was poorer at age 5 and 10 years compared to peers with the same cleft type without additional conditions. Thus, we rejected the null hypothesis.

### Prevalence and definition of additional conditions

For children in this study with CP, including SP and SHP, the prevalence of additional conditions was 40%, which falls within the range presented in the literature (34–52%) [9, 12, 15, 17]. However, for children with CLP, including cleft types UCLP and BCLP, the prevalence range of additional conditions reported in the literature was higher (24–37%) [12, 14, 15] than the prevalence of 17.3% reported in the present study.

The definition of which conditions should be categorized as CP ± L + could be discussed, and different criteria are used in different studies [13]. This, and the fact that different methodologies and availability of techniques and resources to assess genetic and medical conditions differ around the world and over time, contribute to the differences in reported additional conditions in children with CLP [13]. Of the 496 children meeting the inclusion criteria for this study, 169 (34%) had CP ± L +, in this study defined as having a diagnosed syndrome and/or additional malformations and/or intellectual disabilities, resulting in a proportion in accordance with previous literature [14]. After excluding children who did not have registered speech assessments at both 5 and 10 years, 404 children remained, and of these, 118 (29%) presented with CP ± L +. This reflects, to some extent, attrition due to lack of assessable speech [26]. In addition, Aspelin and colleagues established that additional conditions are underreported when comparing data from the CLP registry with medical records and noted that higher rates of additional conditions were registered later in life in the BCLP and UCLP groups than in the CP group, where most additional conditions were already diagnosed at birth [47].

With respect to Robin sequence, the proportion of cases with an isolated RS of 79.2% in the present study was higher compared to earlier reports, ranging from 45–72%, and lower for both syndromic RS (10.4%) compared to 22–28% and RS plus (10.4%) compared to 33% reported in other studies including larger samples [22, 25]. Again, underreporting of additional conditions might explain a larger proportion of isolated RS cases [13], given that RS is already evident early in life, and reporting additional, later diagnosed conditions into the CLP registry can be missed [47].

Other patient-related variables often underreported in the CLP registry, such as neurodevelopmental disorders often diagnosed later in childhood, could also affect the results given the significantly increased risk for children with orofacial clefts to be diagnosed with neurodevelopmental disorders [48]. Furthermore, children born with orofacial clefts in Sweden are not routinely seen by a geneticist. To conclude, different genetic testing standards and resources over time influence findings, and “…many opportunities for misclassification exist” [13] page 3.

### Cleft types, additional conditions, and speech outcomes

It should be considered that differential diagnostics between SP and SHP may be a clinical challenge, and differences in the proportions of SP and SHP between Swedish CLP centers have previously been reported [40]. Despite this, we kept the division between SP and SHP, as children with SP as a group have been reported to have better speech outcomes than children with SHP [26, 40]. At 5 years of age, a significantly lower proportion of children born with SP + and SHP + had age-appropriate consonants than did peers with the same cleft type but without additional conditions. Even at 10 years of age, the SHP + group had significantly lower proportions of age-appropriate consonants than did the SHP- group. Among all cleft types, the type with the poorest speech outcome is BCLP according to both the literature [e.g., [26, 27, 49] and the results of our study. This might have contributed to the somewhat unexpected finding of poorer results regarding some speech outcome measures at some ages in the BCLP- group than in the BCLP + group, although the differences were not significant. The explanation for this discrepancy seems not straightforward but is rather suspected to be related to patient-related variables, some of which we have included and some of which we have no information about because they are not included as variables in the CLP registry.

For the outcome measure without nonoral errors, no significant differences were observed between subgroups with and without additional conditions; however, for all cleft types except BCLP, children without additional diagnoses seemed to have slightly better results. A relatively small percentage of children displayed nonoral errors, which might have contributed to the fact that no significant differences were observed. A significantly lower proportion of the 5-year-olds in the SHP + group than in the SHP- group had sufficient velopharyngeal function. At 10 years of age, the only significant difference observed in velopharyngeal function was a lower proportion in the SP + group than in the SP- group. These findings are in line with an earlier, longitudinal study on speech in children with cleft palate only, which concluded that persistent velopharyngeal incompetence is mostly found in children with additional conditions [21]. 

There were no significant differences between children with UCLP and BCLP with and without additional conditions in this study’s outcome measures, age-appropriate consonant production*,* without nonoral errors, and sufficient velopharyngeal function*.* On the other hand, we cannot rule out that significant differences had been distinguished if the proportion of children with additional conditions had been higher in these cleft subgroups. For example, a close to significantly lower proportion of participants with age-appropriate consonant production were found in the UCLP + group than in the UCLP- group at age 5. Since the statistical power was low in the groups with UCLP and BCLP, it is difficult to interpret non-significant findings with certainty. Overall, the proportion of children with speech outcomes within the expected range was higher for children without additional conditions, except for those in the BCLP group.

### Other variables that may affect speech outcomes

Notably, several other patient-related variables beyond additional conditions have been reported to impact speech outcomes, including sex, cleft type, cleft extent [49] and width [50], ethnicity, socioeconomic status [49], and hearing [7]. In addition, speech outcomes may reflect both the methods and the timing of surgical interventions as well as other care-related factors not registered in the CLP registry, such as surgical skill, the organization of cleft care [40], and the type of speech therapy [51]. In a previous study based on the Swedish CLP registry, completed palatal surgery after 25 months of age had a negative impact on speech at 5 years of age [40]. In our study, there were no differences between groups with and without additional conditions regarding age at completed palatal surgery which could explain differences in speech results. We chose to perform comparisons of cleft subgroups, and the groups would have been too small if we had taken sex into account as well. Other variables included in the CLP registry that may influence speech results, such as the number of speech-language pathologist visits [51], hearing [7], and diagnosed language impairment have shown to be less reliable. We therefore refrained from presenting data on these variables.

### Caregiver-reported outcomes

Patient- and, for younger children, caregiver-reported outcomes are essential in patient-centred healthcare [35]. The high rate of missing data on parental intelligibility ratings [36] in the CLP registry is therefore problematic. Agreement between speech-language pathologists’ ratings of intelligibility and the Intelligibility in Context Scale [36] has been reported to be good for Swedish-speaking 5-year-olds [27]. On the other hand, the suitability of the Intelligibility in Context Scale as a measure of intelligibility has been questioned because of a lack of correlation with intelligibility reference scores; however, this scale is recommended for use by speech and language pathologists to provide an overview of parents' view of their child's ability to communicate [35, 46]. In the present study, there were significantly lower proportions of children with always intelligible speech at 10 years of age in the SP + group and the SHP + group than in the SP- and SHP- groups. This is important information that strengthens results of the perceptual assessment.

### Secondary speech improving surgery and speech language therapy

There were no differences in the cleft subgroups with and without additional conditions regarding the proportions of children who had their first secondary speech improving surgery before 5 years of age or between 5 and 10 years of age. This indicates that children with assessable speech were equally treated, regardless if they had additional syndromes or not.

Children with SP + were significantly more often enrolled in speech language therapy before 5 years of age and between 5 and 10 years of age than were children with SP-. This finding positively reflects the impact of speech language therapy in the SP + group, with no significant differences in age-appropriate consonants at 10 years compared with the SP- group. A recent meta-analysis evaluating changes in the PCC after speech language therapy concluded that children under the age of six are especially likely to achieve a clinically relevant degree of speech improvement [51].

### Limitations

The CP ± L population is small; thus, data must be collected for a long period of time to obtain a larger set of children. This implies difficulties keeping possibly influencing factors constant, such as the material and methods used for data collection. The Swedish CLP registry enables continuous quality assurance of CLP care and intercenter studies with larger datasets. Although the CLP registry has a high degree of coverage and reporting [19, 40] and registered data for the speech variables have been checked for reliability [37–39], when interpreting the results, it is important to be aware that the data are retrospective. This entails certain limitations. For example, potential effect of surgical technique and skill on speech could not be investigated, since no such information were recorded in the CLP registry. Associations have previously been found between a higher number of palatal repairs performed by the surgeons and a better speech outcome [52]. In a previous study based on the CLP registry, the average annual CLP case load 2009 to 2014 varied between centers from 15.7 and 40.8, and the number of chief operators from 1 to 4 [40]. No association was seen between speech outcomes and the number of children with CLP at different centers [40]. This may be partly due to surgeons in centers with fewer cases compensating for this by participating in foreign exchanges to increase their surgical experience.

Also, detailed information on specch therapy was lacking. Furthermore, three different word tests were used for the analysis of speech outcomes during the decade the participants were followed. Since all word tests are designed according to the same principles, with the aim of capturing speech errors that occur with cleft palate [44], we consider the differences between the tests to be of minor importance. However, this is an illustrative example of obstacles when performing longitudinal studies using registry data.

A higher proportion of children with additional conditions than without had missing registrations or missing speech assessments. This could be due to either lack of assessable speech or difficulties participating during speech registration possibly as a result of additional conditions. Thus, the children who had the most severe additional diagnoses were likely excluded. Had these participants’ results been included into the analysis, scored as zero, the results for the subgroups with additional conditions had been poorer. Exclusion of the most affected individuals likely biased group comparisons.

The study was based entirely on data from the Swedish CLP registry with a high-quality, standardized dataset. In Sweden, all children with CLP are treated within the publicly funded healthcare system and have access to multidisciplinary CLP care. The results may therefore have limited applicability to countries with differing systems for CLP care, speech therapy access, and diagnostic protocols. In large parts of the world, healthcare is privately funded and access to multidisciplinary CLP care varies.

## Conclusions

The results add to earlier longitudinal findings, with improvement of speech between 5 and 10 years of age for all cleft groups and, in this study, were also found in children with additional conditions. However, poorer speech, measured as age-appropriate consonants and sufficient velopharyngeal function, was observed in the subgroups with SP + and SHP + at 5 and/or 10 years of age than in the groups without additional conditions. This was also reflected in the caregiver-reported outcome of functional intelligibility at 10 years of age. No differences were seen between children with and without additional conditions regarding secondary speech improving surgery. However, children with SP + received more speech language therapy than peers with SP- both before age 5 and between ages 5 and 10, with positive outcomes at the group level. This study highlights the need for clinical speech-language pathologists to consider the heterogeneity within cleft subgroups and to evaluate every individual’s patient- and care-related factors when planning speech improving interventions.

## Data Availability

The datasets generated and/or analysed during the current study are not publicly available due to ethical restrictions but are available from the corresponding author upon reasonable request. Additionally, to access the data in the Swedish CLP registry, approval from an Ethics Board and Region Skåne is needed (https://www.skane.se/om-region-skane/forskning/for-dig-som-forskar/personuppgifter-och-patientdata/kvb-ansokan-for-utlamnande-av-patientdata/). Lists of included variables and results published in annual reports may be retrieved in Swedish from the website of the CLP registry: http://lkg-registret.se/. An overview of the included data can be provided by the Swedish Research Council: https://www.registerforskning.se/en/registers-in-sweden/easier-to-find-register-data-with-the-register-utiliser-tool/.

## Abbreviations

BCLP: Bilateral cleft lip and palate
CLP: Cleft lip and palate
CP: Cleft palate
CP ± L: Cleft palate with or without cleft lip
PCC: Percentage of consonants correct
RS: Robin sequence
RS plus: Robin sequence with associated conditions but no identifiably specific syndrome
SHP: Cleft soft and hard palate
SP: Cleft soft palate
SVANTE: The Swedish articulation and nasality test
TOPS: Timing of Primary Surgery for Cleft Palate
UCLP: Unilateral cleft lip and palate
VPC-R: Velopharyngeal competence rating
VPF: Velopharyngeal function
+: With syndromes and/or additional malformations and/or intellectual disability
-: Without known syndromes, additional malformations, and intellectual disability
